# Systematic Review of TST Responses in People Living with HIV in Under-Resourced Settings: Implications for Isoniazid Preventive Therapy

**DOI:** 10.1371/journal.pone.0049928

**Published:** 2012-11-27

**Authors:** Andrew D. Kerkhoff, Katharina Kranzer, Taraz Samandari, Jessica Nakiyingi-Miiro, Christopher C. Whalen, Anthony D. Harries, Stephen D. Lawn

**Affiliations:** 1 School of Medicine and Health Sciences, The George Washington University, Washington D.C., United States of America; 2 Desmond Tutu HIV Centre, Institute of Infectious Disease and Molecular Medicine, Faculty of Health Sciences, University of Cape Town, Cape Town, South Africa; 3 Department of Clinical Research, Faculty of Infectious and Tropical Diseases, London School of Hygiene and Tropical Medicine, London, United Kingdom; 4 Division of Tuberculosis Elimination, Centers for Disease Control and Prevention, Atlanta, Georgia, United States of America; 5 Medical Research Council/Uganda Virus Research Institute, Uganda Research Unit on AIDS, Entebbe, Uganda; 6 Department of Epidemiology and Biostatistics, College of Public Health, University of Georgia, Athens, Georgia, United States of America; 7 International Union Against Tuberculosis and Lung Disease, Paris, France; McGill University, Canada

## Abstract

**Background:**

People living with HIV (PLWH) who have positive tuberculin skin tests (TST) benefit from isoniazid preventive therapy (IPT) whereas those testing TST-negative do not. Revised World Health Organization guidelines explicitly state that assessment of TST is not a requirement for initiation of IPT. However, it is not known what proportions of patients will benefit from IPT if implemented without targeting according to TST status. We therefore determined the proportions of PLWH who test TST-positive.

**Methodology/Principal Findings:**

We systematically reviewed the literature published between January 1990 and February 2012 to determine the proportions of patients without active tuberculosis attending HIV care services in low and middle-income countries who tested TST-positive (≥5 mm induration). Proportions were also determined for different CD4 count strata. Data from 19 studies with 9,478 PLWH from sub-Saharan Africa, Asia and Central and South America were summarized. The vast majority were not receiving antiretroviral therapy (ART). A sub-analysis was conducted of 5 studies (5,567 subjects) from high TB prevalence countries of PLWH with negative TB screens attending HIV care and treatment settings for whom CD4 stratified data were available. The median proportion of PLWH testing TST-positive overall was 22.8% (range, 19.5–32.6%). The median (range) proportions with CD4 cell counts of <200, 200–499 or ≥500 cells/µL who tested positive were 12.4% (8.2–15.3%), 28.4% (20.1–36.9%) and 37.4% (31.3–56.3%), respectively. Heterogeneity in the data precluded calculation of pooled summary estimates.

**Conclusions/Significance:**

In most settings, if IPT is administered to PLWH pre-ART without assessment of TST status, only a minority of those treated are likely to benefit, especially among those with the lowest CD4 cell counts. This may be inefficient use of resources and cost-effectiveness analyses should take this into account. Local knowledge of TST response rates may help inform policies. New simple means of identifying those who will benefit from IPT are needed to permit appropriate targeting of this intervention.

## Introduction

HIV-associated tuberculosis (TB) remains a substantial challenge to global health [1], accounting for an estimated 12% (1.1 million) of the overall TB caseload and one quarter (0.35 million) of HIV/AIDS deaths worldwide [2]. Four main programme interventions are recommended to prevent HIV-associated TB [3]. These include scale-up of antiretroviral therapy (ART) [4] used in combination with intensified case finding, isoniazid preventive therapy [IPT] and infection control, the latter interventions being referred to by the acronym the ‘three I’s strategy’ [5]. However, a large majority of cases are in sub-Saharan Africa where the capacity to deliver the needed control interventions is limited [6]. For many years, implementation of IPT in particular has been very poor [7].

A meta-analysis of placebo-controlled trials reported that IPT in people living with HIV (PLWH) conferred an overall risk reduction of 33% (95%CI, 13–49%) [8]. A beneficial effect was only observed among those who tested tuberculin skin test (TST) positive in whom there was a substantially greater risk reduction of 64% (95%CI, 39–78%). In contrast, no benefit was observed among PLWH (n = 2,490) in these trials with negative or anergic TST reactions [8]. Based on this evidence, WHO guidelines between 1998 and 2010 emphasized the main use of IPT as being for prophylaxis of those who are TST-positive [9].

With low uptake of IPT and recognition that the process of TST assessment was a major programmatic stumbling block, the 2011 revision of the WHO IPT guidelines made a strong explicit recommendation that “TST is not a requirement for initiating IPT in people living with HIV” although it “can be used where feasible” [10]. Eliminating the need for assessment of TST status has simplified implementation substantially. However, this means that the intervention is no longer targeted at those who will derive benefit and a small proportion of PLWH who are unlikely to derive benefit might be harmed. The cost-effectiveness of IPT with or without use of TST assessment in a clinical population is likely to be strongly associated with the prevalence of positive TST tests. We therefore conducted this systematic review.

The proportion of PLWH testing TST-positive is likely to be associated with the prevalence of TB in the local community, the prevailing force of infection, social mixing patterns and the clinical population sampled. Prevalence also increases with age [11] but may be attenuated in those with suppression of cell-mediated immune responses. Progressive CD4 cell count loss that characterizes HIV progression is strongly associated with increasing proportions of anergic TST responses [12]. We therefore conducted this systematic literature review and meta-analysis to determine the proportion of PLWH in HIV care and treatment settings who test TST positive and how this proportion varies across a range of CD4 cell count strata.

## Methods

### Systematic Search Strategy

We conducted the search and review process according to a predefined protocol and the study conformed to the PRISMA statement checklist [13]. Embase, Global Health, Medline and Web of Science were searched for potentially relevant citations using a defined search strategy (Table S1). The search included articles published in English between January 1st 1990 and February 5th 2012, covering the majority of the epidemic [14]. Initial searches found no relevant data published before this period. The bibliographies of review articles on interferon-γ-release assays were specifically searched for additional articles. Abstracts from the World Conference on Lung Health of the International Union Against Tuberculosis and Lung Disease (IUATLD) published between 2004 and 2011 were searched by hand for additional relevant citations.

Citations identified through the search process were compiled into Endnote X4 and duplicates removed. Titles and abstracts were screened for eligibility according to pre-defined inclusion and exclusion criteria and full-texts were reviewed for eligibility by ADK and SDL. The authors of eligible studies were contacted as required to clarify or modify the stratification of their data by CD4 cell count.

A study was eligible for inclusion if it was conducted in a low-income or middle-income country (as defined by the World Bank list of economies current on 1st July 2011) [15]; the study subjects were PLWH aged 15 years or older; the study reported TST results (positive if ≥5 mm induration) for individuals stratified into at least two CD4 cell count strata (CD4 cell counts <200 and ≥200 cells/µL); data were reported on PLWH without evidence of current active TB disease, and if the study contained 50 or more subjects meeting the above criteria. If two eligible studies presented overlapping samples, only the study with a larger sample size was included. Studies using a two-step TST-testing were excluded as this methodology is not used in public health programmes in resource-limited settings.

Data from eligible studies were abstracted directly into a structured Microsoft Excel spreadsheet. Study characteristics recorded included title, authors, year of publication, study period, study location, mean or median patient age, bacille Calmette et Guerin (BCG) scar status, clinical setting, ART status, TST methodology, inclusion of special populations or sub-groups and the total number of PLWH with TST-positive and TST-negative results stratified by CD4 counts (either using <200 and ≥200 cells/µL or using <200, 200–499 and ≥500 cells/µL).

### Sub-analysis of Studies

For inclusion in a sub-analysis and potential meta-analysis, we selected those studies in high TB prevalence settings which were of sufficient size, were conducted among PLWH accessing HIV care and treatment services and in whom active TB had been excluded. This selection reflects the PLWH who are specified in WHO guidelines as being the key targets for IPT [10]. High TB prevalence was defined as an average ≥100 cases per 100,000 population using WHO estimates for the five-year period spanning the study mid-point [7]. A minimum of 40 PLWH were required in each CD4 cell count stratum to limit imprecision around estimates (anticipated range, 10%–50% testing TST-positive). Studies evaluating TB diagnostic assays or conducted in TB clinics were excluded due to the likelihood for selection bias during enrolment.

Two reviewers (ADK and SDL) assessed the quality of studies using a graded checklist (Table S2). A study was scored “good quality” if it received 70% or more of available points, “medium quality” if it received between 50–69% and “lower quality” if less than 50%. The primary outcome of interest for our study was the proportion of PLWH who tested TST-positive among those in each of the following CD4 cell count strata: <200, 200–499 and ≥500 cells/µL. Risk of bias in individual studies and across studies was also assessed.

### Data-analysis

All statistical analyses were conducted using STATA 10. The proportions of PLWH testing TST-positive were calculated with corresponding 95% confidence intervals (95% CI). These data were stratified by geographic region, country TB prevalence (<100, 100–249 or ≥250 cases per 100,000 population) and CD4 cell count (<200 and ≥200 cells/µL or <200, 200–499 and ≥500 cells/µL). Findings were presented using forest plots. The median and corresponding range of proportions of patients testing TST-positive were calculated. I-squared statistics [16] were calculated to assess the heterogeneity of the data stratified by CD4 cell count.

## Results

### Studies Included

**Figure 1:**
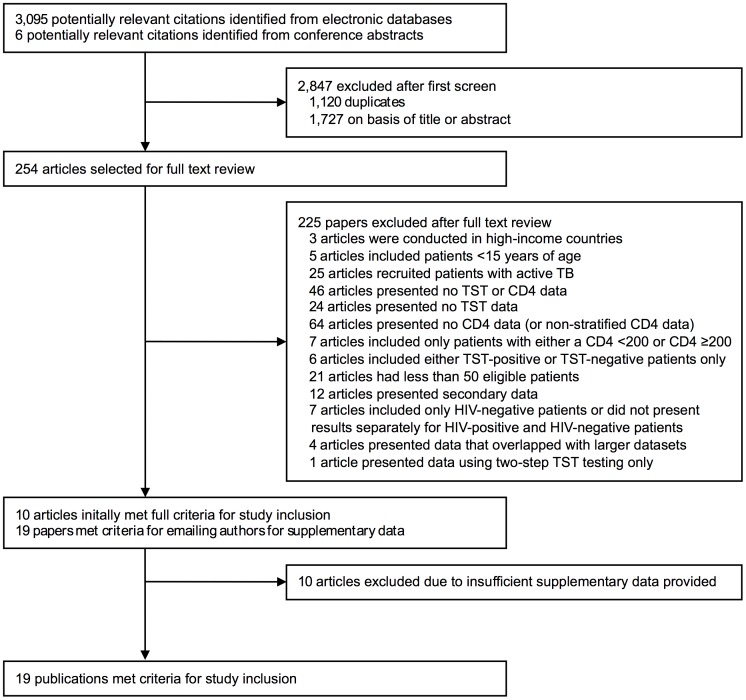
Selection of eligible studies containing data on the proportion of people living with HIV testing tuberculin skin test (TST)-positive stratified by blood CD4 cell count.

Of 3,095 potentially relevant citations identified, 254 were selected for full-text review (Figure 1 **)**. Ten publications initially met inclusion criteria and nine others met inclusion criteria after supplementary data were provided by study authors. Ten others remained ineligible despite seeking supplementary data (Table S3). The final group of eligible publications (n = 19) were published between 1997 and 2011.

Of the studies included (n = 19), five were from sub-Saharan Africa, four from South-East Asia, four from the Americas, three from the Eastern Mediterranean two from the Western Pacific and one from Europe (Table 1). A majority (n = 14; 74%) were conducted in high-prevalence TB countries (estimated TB prevalence ≥100 cases per 100,000 population). The mean or median age of study subjects ranged between 22 and 40 years, but overall 90% of patients were from studies in which the mean or median age was within the range 29–40 years. The methods used for excluding active TB were reported by 13 (68%) studies but were not standardised. All studies defined TST positivity using a cut-off of ≥5 mm diameter of induration. Study quality was assessed as high in six studies, medium quality in eight and lower quality in five. Risks of bias in reporting the outcome of interest or publication bias were considered to be low.

**Table 1:** Characteristics of studies presenting data on the proportion of people living with HIV who tested TST-positive stratified by CD4 cell counts.

Study	Country	Study years	Five year average TB prevalence rate (per 100,000)	Study setting	Eligible patients (N)	Mean age (years)	Study population	Special populations	Methods used to exclude active TB among study subjects	TST methods	Patients on ART at enrolment (%)	Patients with at least one BCG scar (%)	Included in meta-analysis
AFRICA													
Lugada et al (2002) [44]	Uganda (Entebbe)	1995–1999	227.0	Semi-urban	2001	32.3	Individuals living within 15 km of community HIV care clinics	No WHO stage 4 patients were included	Symptom screen, clinical examination, chest X-ray	5-TU PPD	0	50.7	Yes
Rangaka et al (2007) [45]	South Africa (Khayelitsha)	…	654.5	Urban	57	31	Individuals attending an integrated HIV-TB clinic for HIV testing	…	Symptom screen, clinical examination, Karnofsky score <60	2-TU PPD RT23	0	51.0	No
Karam et al (2008) [46]	Senegal (Dakar)	2003–2005	462.0	Urban	274	37*	Individuals attending an infectious disease department or ambulatory care centre of a hospital	Only patients with an HIV diagnosis in the previous three months	Clinical examination, chest X-ray or microbiological evidence or Karnofsky score <80	2-TU PPD RT23 and read after 48–72 hours	0	72.6	Yes
Oni et al (2011) [47]	South Africa (Khayelitsha)	2008–2010	785.0	Urban	238	30*	Individuals attending an integrated HIV-TB clinic pre ART initiation	…	Symptom screen, chest X-ray (if TST+) and sputum smear and culture	2-TU PPD RT23 and read after 48–72 hours	0	54.0	No
Samandari et al (2011) [21]	Botswana (Gaborone and Francistown)	2004–2009	750.0	Urban	1891	35	Individuals attending government clinics providing ART and IPT services	…	Symptom screen and chest X-ray	5-TU PPD RT23 and read after 48–72 hours	2.0	78.0	Yes
AMERICAS													
Garcia-Garcia et al (2000) [48]	Mexico (Mexico City)	1992–1993	123.0	Urban	801	31.0	Individuals requesting an HIV test at an HIV testing centre	72.9% of patients were MSM and 1.1% were IDU	Clinical screen, chest X-ray, sputum smear and culture	5-TU PPD RT23 and read after 48–72 hours	12.4	81.5	Yes
Miranda et al (2007) [49]	Brazil (7 states)	1995–2001	104.0	…	98	34*	Individuals attending a public HIV treatment facility	59% of patients were MSM, 13% had a history IDU and 7% had a history of incarceration	Methods unclear	…	84.0	…	No
Balcells et al (2008) [50]	Chile (Santiago)	2006–2007	16.5	Urban	109	38.8*	Individuals attending HIV outpatient clinics	Only patients with CD4 count >100 were included	Symptom screen	2-TU PPD RT23 and read after 48–72 hours	57.8	83.5	No
Moura et al (2011) [51]	Brazil (Recife)	2007–2010	59.0	Urban	864	40.4	Individuals in outpatient hospital services that serve as HIV/AIDS referral services	…	…	0.1 ml PPD RT23 and read after 72 hours	77.7	…	No
EASTERN MEDITERRANEAN													
Davarpanah et al (2009) [52]	Iran (Shiraz)	2008	36.0	Urban	152	38*	Individuals attending an infectious disease clinic	…	…	5-TU PPD and read after 48–72 hours	…	…	No
Alavi et al (2010) [53]	Iran (Ahvaz)	2008	36.0	Urban	62	30.5 and 34.3	Randomly selected individuals in prison and attending drug addiction centres	Only drug addicted individuals were included	Methods unclear	0.1 ml 5-TU PPD (Razi Institute) and read after 48–72 hours	…	…	No
Mardani et al (2010) [54]	Iran (Tehran)	2007	37.7	Urban	50	39	Individuals attending an HIV clinic	…	Methods unclear	0.1 ml PPD and read after 48 hours	24.0	100	No
EUROPE													
Vitek et al (2009) [55]	Russia (Orel Oblast)	2004	178.0	…	150	28	Individuals adherent to previous follow-up visits at an AIDS centre	61% of patients were IDU and 17.3% had a history of imprisonment	…	2-TU Russian tuberculin (Research Institute of Vaccines and Antitoxins) and read after 48–72 hours	…	78.0	No
SOUTH-EAST ASIA													
Yanai et al (1997) [56]	Thailand (Chiang Rai)	1992–1994	208	Urban	85	28.7 and 22.3	Individuals attending a blood bank and female sex workers attending STD clinic	62.7% of patients were blood donors and were 37.3% female sex workers	…	5-TU Tubersol PPD (Connaught Laboratories) and read after 48–72 hours, but up to 5 days	…	72.4 and 62.0	No
Hiransuthikul et al (2003) [57]	Thailand (Bangkok)	1994–1996	201.0	Urban	160	29*	Individuals attending an AIDS clinic	Only asymptomatic and early symptomatic patients were included. 37.5% of patients were homosexual or bisexual and 19.3% were commercial sex workers	Symptom screen, chest X-ray and sputum microscopy or Karnofsky score <70	5-TU PPD-S and read after 48–72 hours	…	…	Yes
Gupta et al (2007) [58]	India (Pune)	2002–2005	284.0	Urban	752	23*	Pregnant women attending a public hospital	Only pregnant women were included	Symptom screening: if positive, chest x-ray, sputum smear and culture	5-TU PPD and read after 48–72 hours	…	…	No
Swaminathan et al (2008) [59]	India (Chennai and Madurai)	2000–2005	248.0	Urban	158	31	Individuals attending government-funded TB clinics	…	Symptom screen, chest X-ray and 3 sputum cultures	1-TU PPD RT23 and read after 48–72 hours	…	48.3	No
WESTERN PACIFIC													
Jiang et al (2009) [60]	China (Yunnan Province)	…	146.7	…	68	33.9 and 33.7	Individuals from within a province	…	Chest X-ray, sputum microscopy and sputum culture	5-TU PPD RT23 and read after 48–72 hours	8.8	100	No
Nguyen et al (2011) [61]	Viet Nam (Ho Chi Minh City)	2009–2010	334.4	Urban	369	30*	Individuals attending a public clinic that offers HIV services	62.9% of patients were IDU, 15.5% had a history of incarceration, 44.2% have a history of TB	Symptom screen, chest X-ray, sputum smear and culture	5-TU PPD and read after 48–72 hours	58.3	55.6	No

* Denotes median age.

“…” denotes information not stated.

TU = tuberculin units; PPD = purified protein derivative; STD = sexually transmitted disease; MSM = men who have sex with men;

IDU = people who inject drugs; ART = antiretroviral therapy; IPT = isoniazid preventive therapy.

The proportions of PLWH receiving ART at the time of TST assessment was reported by ten studies and two other studies pre-dated national ART implementation and these were therefore assumed to be ART-naïve populations. Among these twelve studies, the proportion of patients receiving ART ranged from 0 to 84% with a median of 5.4% (IQR, 0–32.5%).

The studies included a total of 9,478 subjects and 2,820 (29.8%) of these had CD4 cell counts <200 cells/µL and 6,658 (70.3%) had counts of >200 cells/µL. In 16 studies the data were further stratified, providing data from 4,034 subjects with CD4 counts of 200–499 cells/µL and 2,231 with counts of ≥500 cells/µL.

### Proportions Testing TST-positive

**Figure 2:**
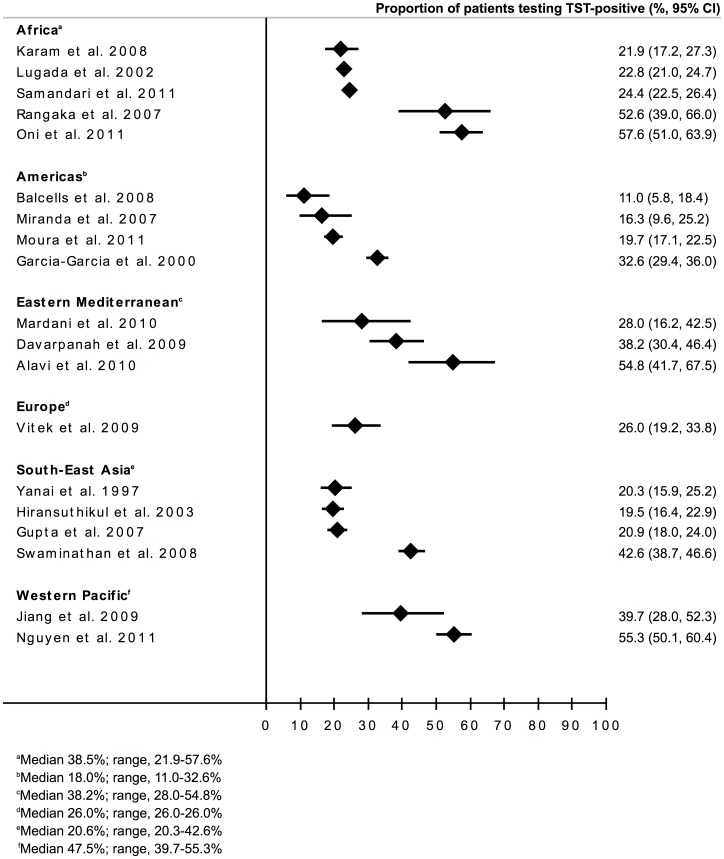
Forest plot showing the proportions (%, 95%CI) of people living with HIV testing TST-positive in the all studies (n = 19) with data grouped according to: a) geographical region; b) country TB prevalence (<100, 100–249 and ≥250 cases per 100,000 population) and c) CD4 cell count (<200 or ≥200 cells/µL).

The numbers and proportions of PLWH testing TST-positive for each of the 19 studies are summarized (Table 2 **)**. The overall median proportion testing TST positive was 26.0% (IQR, 20.6–41.2%; range 11.0–57.6%). The proportions testing positive stratified according to geographical region (Figure 2A) and country TB prevalence (Figure 2B) were displayed in forest-plots. The data were found to be heterogeneous and no clear associations with either of these two variables were evident.

**Table 2:** The proportion of people living with HIV who tested TST-positive stratified by CD4 cell count categories <200, ≥200, 200–499 and ≥500 cells/µL.

Study	All study subjects		CD4 count <200 cells/µL		CD4 count ≥200 cells/µL		CD4 count 200–499 cells/µL		CD4 count ≥500 cells/µL	
	No. with TST results	Positive TST n (%)	No. with TST results	Positive TST n (%)	No. with TST results	Positive TST n (%)	No. with TST results	Positive TST n (%)	No. with TST results	Positive TST n (%)
AFRICA										
Lugada et al (2002) [44]	2001	457 (22.8)	931	115 (12.4)	1070	342 (32.0)	647	184 (28.4)	423	158 (37.4)
Rangaka et al (2007) [45]	57	30 (52.6)	8	2 (25.0)	49	28 (57.1)	31	17 (54.8)	18	11 (61.1)
Karam et al (2008) [46]	274	60 (21.9)	146	12 (8.2)	128	48 (37.5)	85	25 (29.4)	43	23 (53.5)
Oni et al (2011) [47]	238	137 (57.6)	51	25 (49.0)	187	112 (59.9)	137	79 (57.7)	50	33 (66.0)
Samandari et al (2011) [21]	1891	462 (24.4)	575	88 (15.3)	1316	374 (28.4)	952	250 (26.3)	364	124 (34.1)
AMERICAS										
Garcia-Garcia et al (2000) [48]	801	261 (32.6)	309	47 (15.2)	492	214 (43.5)	325	120 (36.9)	167	94 (56.3)
Miranda et al (2007) [49]	98	16 (16.3)	35	3 (8.6)	63	13 (20.6)	35	5 (14.3)	28	8 (28.6)
Balcells et al (2008) [50]	109	12 (11.0)	13	1 (7.7)	96	11 (11.5)	73	4 (5.5)	23	7 (30.4)
Moura et al (2011) [51]	864	170 (19.7)	137	13 (9.5)	727	157 (21.6)	386	70 (18.1)	341	87 (25.5)
EASTERN-MEDITERRANEAN										
Davarpanah et al (2009) [52]	152	58 (38.2)	35	8 (22.9)	117	50 (42.7)				
Alavi et al (2010) [53]	62	34 (54.8)	12	6 (50.0)	50	28 (56.0)				
Mardani et al (2010) [54]	50	14 (28.0)	14	2 (14.3)	36	12 (33.3)	33	10 (30.3)	3	2 (66.7)
EUROPE										
Vitek et al (2009) [55]	150	39 (26.0)	10	2 (20.0)	140	37 (26.4)	79	15 (19.0)	61	22 (36.1)
SOUTH & SOUTH-EAST ASIA										
Yanai et al (1997) [56]	311	63 (20.3)	85	14 (16.5)	226	49 (21.7)				
Hiransuthikul et al (2003) [57]	600	117 (19.5)	160	18 (11.3)	440	99 (22.5)	344	69 (20.1)	96	30 (31.3)
Gupta et al (2007) [58]	752	157 (20.9)	57	11 (19.3)	695	146 (21.0)	360	77 (21.4)	335	69 (20.6)
Swaminathan et al (2008) [59]	631	269 (42.6)	158	64 (40.5)	473	205 (43.3)	313	138 (44.1)	160	67 (41.9)
WESTERN PACIFIC										
Jiang et al (2009) [60]	68	27 (39.7)	9	1 (11.1)	59	26 (44.1)	28	10 (35.7)	31	16 (51.6)
Nguyen et al (2011) [61]	369	204 (55.3)	75	31 (41.3)	294	173 (58.8)	206	116 (56.3)	88	57 (64.8)

We next summarized the data stratified according to CD4 cell count strata (Table 2). These data stratified by CD4 cell counts of <200 cells/µL and ≥200 cells/µL were available for all 19 studies included and are displayed in Figure 2C. Although there was heterogeneity within these groups, the proportions testing TST-positive tended to be higher among PLWH with CD4 cell counts of ≥200 cells/µL (median 33.3%; IQR, 22.1–43.8%; range, 11.5–59.9%) compared to those with CD4 cell counts <200 cells/µL (median 15.3%; IQR, 11.2–23.9%; range, 7.7–50.0%).

### Sub-analysis of Studies

Of the 16 studies that provided TST data stratified by three CD4 cell count strata (<200, 200–499 and ≥500 cells/µL), 5 also fulfilled the additional criteria for inclusion in the meta-analysis (Table 1). Four studies were scored as high quality and one medium quality and they included 5,567 (58.7%) of the overall 9,478 PLWH in the review. The studies were from Senegal, Uganda, Botswana, Mexico and Thailand and the average five-year national TB prevalence estimates for these countries ranged between 123 and 750 cases per 100,000 population. Mean age ranged between 29 and 37 years. The proportion of PLWH receiving ART at the time of TST assessment was low (range, 0–12.4%) and most had evidence of BCG vaccination (Table 1). Among subjects (n = 5,567) included in the analysis, 2,121 (38.1%) had a CD4 cell count <200 cells/µL, 2,353 (42.3%) a CD4 cell count 200–499 cells/µL, and 1,093 (19.6%) a CD4 cell count ≥500 cells/µL.

**Figure 3:**
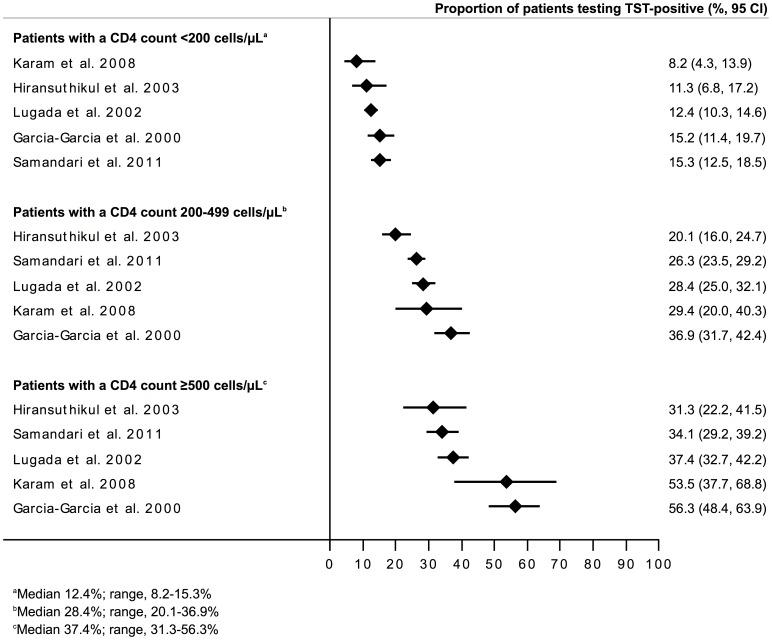
Forest plot showing the proportions (%, 95%CI) of people living with HIV testing TST-positive in studies (n = 5) included in the meta-analysis with data grouped according to CD4 cell count strata (<200, 200–499 and ≥500 cells/µL). The median proportions testing positive in these three groups were 12.4% (range, 8.2–15.3%), 28.4% (range, 20.1–36.9%) and 37.4% (range, 31.3–56.3%). The I^2^ statistics for these three groups of data were 54.4% (P = 0.067), 84.2% (P<0.001) and 86.8% (P<0.001), respectively.

The median proportion testing TST-positive in these 5 studies was 22.8% (range, 19.5–32.6%). These data are shown in a forest plot stratified by CD4 cell count <200 cells/µL, 200–499 cells/µL and ≥500 cells/µL (Figure 3). The proportions testing TST-positive differed between the three groups, with medians of 12.4% (range, 8.2–15.3%), 28.4% (range, 20.1–36.9%) and 37.4% (range, 31.3–56.3%), respectively. I-squared statistics showed significant heterogeneity within each stratum, however, precluding the calculation of pooled summary estimates.

## Discussion

In this systematic review, we summarized data from 19 studies that included 9,478 PLWH from low- and middle-income countries in sub-Saharan Africa, Asia and Central and South America. The median proportion of PLWH testing TST-positive was 26.0% overall but with substantial heterogeneity (range, 11.0–57.6%). Five of these studies (5,567 subjects) were selected for inclusion in a sub-analysis on the basis of high country TB prevalence, adequate study size and quality and inclusion of PLWH without TB attending HIV care and treatment settings. Among these studies, the median proportions of patients with CD4 cell count <200 cells/µL, 200–499 cells/µL and ≥500 cells/µL testing TST-positive were 12.4% (range, 8.2–15.3%), 28.4% (range, 20.1–36.9%) and 37.4% (range, 31.3–56.3%), respectively. Data heterogeneity, however, precluded calculation of pooled summary estimates. These data indicate that in most settings, implementation of IPT among PLWH without assessment of TST status is likely to benefit a minority of patients across all CD4 count categories.

In resource-limited settings, assessment of TST status is the only recommended means of identifying patients who are likely to benefit from IPT [10]. However, this unfortunately presents a substantial operational barrier to implementation. The test requires a refrigerated supply of purified protein derivative and sterile supplies. Health care workers must be trained to administer the test and accurately read the result. In order to reduce loss to follow-up after TST placement [17] such novel interventions as training of community outreach auxiliaries in the reading of the induration must be studied. Although interferon-γ-release assays give fewer false-positive results, there is no evidence that these are any more predictive of IPT benefit and these tests are not recommended for use in resource-limited settings [10], [18].

Removal of the requirement for TST assessment simplifies implementation of IPT and may thereby help catalyze scale-up of IPT. However, this study suggests that overall only approximately 1 in 4 PLWH stand to benefit from IPT if implemented in an untargeted way. Although this would save resources needed to assess TSTs, this would be wasteful of both patient and scarce health care resources used in treating and following-up large numbers of PLWH who would derive no benefit. Cost-effectiveness analyses suggest that use of IPT either in a targeted or untargeted way is cost-effective compared to not implementing IPT at all [19], [20]. In South Africa, both strategies were estimated to be equally cost-effective [20] whereas in Uganda targeting IPT by TST status was more favourable in terms of incremental cost per QALY gained [19]. Both studies used optimistic assumptions about the duration of the benefit of IPT. As ART is increasingly available in resource-constrained settings, cost effectiveness analyses of IPT – including long-term IPT – in combination with ART are urgently needed to better assess the potential public health utility of these interventions.

Data from southern Africa increasingly suggest that because of high prevailing rates of community TB transmission, benefit from IPT is largely limited to the period within which it is taken [21]–[23]. Thus, long-term IPT is now an option recommended by WHO [10]. If IPT is to be increasingly used on a long-term basis, TST assessment may be even more important as suggested by a cost-effectiveness analysis from Botswana. In this study, provision of IPT for 36 months to TST-positive individuals only was more cost-effective than providing IPT without TST assessment or providing IPT for 6 months only [24].

Concerns have been expressed about the risks of widespread use of isoniazid monotherapy fuelling development of drug resistance. There is no evidence of such an effect from data generated during carefully conducted randomized controlled trials [25], and yet it is unknown what risk would be associated with widespread availability of isoniazid monotherapy within communities outside the context of intervention trials. Since development of antimicrobial resistance is directly related to how much a drug is used, limiting drug exposure to the minority of PLWH who are likely to benefit would be prudent.

Isoniazid is well known to be associated with low rates of hepatotoxicity and occasional deaths [26]. Consistent with previous studies, rates in two large IPT trials in southern Africa have recently reported rates of hepatotoxicity of approximately 0.1–1.0% [27], [28]. Peripheral neuropathy may also be exacerbated by isoniazid and can be disabling and significantly reduce the quality of life. Although overall rates of toxicity are low and mortality rare, these events cannot be ignored and they provide an important argument against use of IPT in those who are TST-negative.

There was great heterogeneity between studies in the proportions testing TST positive. This observation suggests that knowledge of TST responses in specific countries or settings may help local decisions on the pros and cons of targeted or untargeted IPT delivery. This heterogeneity may reflect both the local TB epidemiology as well as study-related factors. The latter may include study design, methodology used for TST assessment (including dose of PPD), the accuracy with which this was done, potential biases associated with non-return of patients for TST assessment, the rigour with which TB was excluded and the characteristics of the patients tested. While we have attempted to broadly summarize these variables, it was not possible to precisely define the role of each. Importantly, however, the mean or median ages of PLWH included in the meta-analysis all fell within a narrow range (29–37 years). It was notable that country TB prevalence did not appear from the forest plots to be a more dominant factor. This may be related to imprecision of TB prevalence estimates, differences between national estimates and prevalence in the specific communities studied or the fact that TB exposure is related to cumulative exposure over the life-time of the individual rather than current prevalence.

The proportions of positive TST results stratified by CD4 cell count observed among the total group of 19 studies were very similar to those found in the 5 studies that were selected for further analysis, providing reassurance that study selection did not introduce substantial bias. Moreover, many of the other studies that were excluded from the systematic review also report that between 22–25% of PLWH tested TST-positive [29]–[33] (Table S3). Although we were unable to include the unpublished data from the THRio study in Brazil, an earlier publication from this study reported that 24.8% of 5,492 PLWH tested TST positive [34]. The proportions testing positive may be higher in South Africa where rates of TB transmission are very high [11]. However, although the two studies identified from South Africa were not eligible for inclusion in the meta-analysis, data from 1,891 PLWH in neighbouring Botswana were included (Table 2).

These data help to rationalize prevention strategies that need to be tailored according to subjects’ CD4 cell counts. As CD4 cell counts decrease during the natural history of HIV infection, risk of TB progressively increases [14]. However, at the same time, the proportion of individuals who are TST-positive and who might benefit from IPT paradoxically decreases. Thus, at low CD4 cell counts, ART is the key intervention needed to reduce the risk of death and to serve as the principal TB preventive intervention. This decreases TB risk by 67% (95%CI, 61–73%) [35], provides benefit regardless of TST status [21] and can be started without the need for careful exclusion of active TB [36]. There is also growing evidence of the TB preventive effect of ART at higher CD4 cell counts >350 cells/µL [37]. During long-term ART, however, TB incidence rates remain several times higher than rates in the community despite good CD4 cell count recovery [38], [39]. Observational data suggest there is an additive effect by using ART and IPT concurrently [21], [34], [35] and this has now been confirmed by data from a randomised controlled trial [40]. ART induces recovery of TST responses [41], [42] and so a greater proportion of PLWH during long-term ART may benefit from IPT compared to the proportion at baseline. Few PLWH in this meta-analysis, however, were receiving ART and so this could not be assessed. Studies defining the impact of IPT according to TST status in patients receiving ART are needed as this may not necessarily be the same as that observed in the pre-ART era [8].

Although it is being recommended that ART be started at increasingly high CD4 cell counts, IPT might be prioritized for PLWH prior to ART eligibility. Ideally HIV would be identified early in the course of disease and all those testing TST-positive would receive a course of IPT prior to becoming eligible for ART. However, even in those with CD4 cell counts of ≥500 cells/µL, less than two in five PLWH (37.4%) tested TST-positive. Use of TST assessment (or a new alternative test) in such patients would permit appropriate targeting of therapy and could substantially reduce the numbers unnecessarily treated.

Strengths of this study include a comprehensive search strategy which identified studies from diverse geographic regions with a range of TB prevalence estimates. Large numbers of PLWH overall and with a broad range of CD4 cell counts were included. Weaknesses include the paucity of data from PLWH receiving ART, the lack of availability of data from other large as yet unpublished studies and the inability to precisely define the impact of other potential variables. In addition, the reliability of TST assessment could not be assessed and the methods for excluding TB were not standardized. Heterogeneity in the data precluded calculation of pooled summary estimates.

In conclusion, we found that a minority PLWH were TST-positive, even in the highest TB prevalence settings, and that the proportion testing positive was strongly associated with CD4 cell counts. This factor may undermine the cost-effectiveness of use of untargeted IPT. Local knowledge of TST response rates may help inform policies. Operational research to improve TST implementation as well as development of new, simple means to identify those who will benefit from IPT are urgently needed [43].

## Supporting Information

Table S1 **Overview of search strategy.**
(DOCX)

Table S2 **Graded quality assessment checklist.**
(DOCX)

Table S3 **Summary of studies meeting criteria for contacting authors for further data but for which insufficient secondary data was provided for study inclusion.**
(DOCX)
