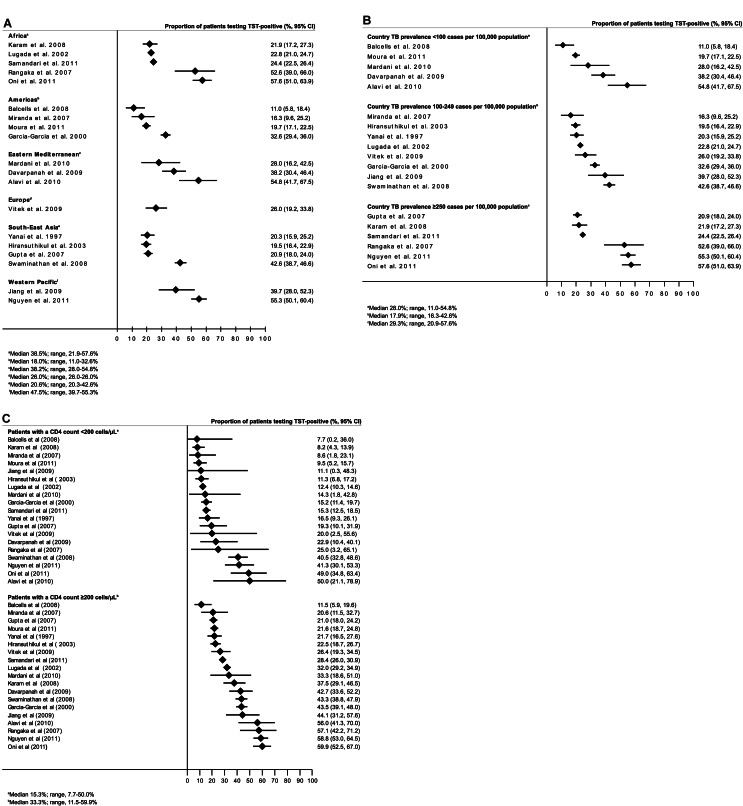# Correction: Systematic Review of TST Responses in People Living with HIV in Under-Resourced Settings: Implications for Isoniazid Preventive Therapy

**DOI:** 10.1371/annotation/e4800fcf-c58a-4d5a-a90a-bb6aed93454a

**Published:** 2013-05-23

**Authors:** Andrew D. Kerkhoff, Katharina Kranzer, Taraz Samandari, Jessica Nakiyingi-Miiro, Christopher C. Whalen, Anthony D. Harries, Stephen D. Lawn

The version of Figure 2 that appeared in the article was incomplete. A complete version of the figure is available here: 

**Figure:**