# Supplementary material for: Systematic Review of TST Responses in People Living with HIV in Under-Resourced Settings: Implications for Isoniazid Preventive Therapy
Source: PLoS One. 2012 Nov 27;7(11):e49928. doi: 10.1371/journal.pone.0049928 (PMC3507950; doi:10.1371/journal.pone.0049928)
Supplement: Table S1 — Overview of search strategy. (DOCX) [file pone.0049928.s001.docx]

**Table S1. Overview of search strategy**

| **Set** | **Embase** | **Global Health** | **Medline** | **Web of Science**** |
| --- | --- | --- | --- | --- |
| 1  2 | TUBERCULIN TEST  (TST or tuberculin adj1 sensitivity adj1 test or tuberculin adj1 skin adj1 test or Mantoux adj1 tuberculin adj1 skin adj1 test or Mantoux adj1 test or Mantoux adj1 screening adj1 test or PPD or purified adj1 protein adj1 derivative or Pirquet adj1 test) | TUBERCULIN and SKIN TESTS | TUBERCULIN TEST | .TI or .TS |
| 4 | Set 1 and 2 were combined with “or” | | | |
| 5  6 | LATENT TUBERCULOSIS  (latent adj1 tuberculosis or LTB or LTBI) |  | LATENT TUBERCULOSIS | .TI or .TS |
| 7 | Set 5 and 6 were combined with “or” | | | |
| 8  9 | ISONIAZID  (Isoniazid or isoniazid adj1 preventive adj1 therapy or IPT or INH) | ISONIAZID | ISONIAZID | .TI or .TS |
| 10 | Set 8 and 9 were combined with “or” | | | |
| 11 | Set 4, 7, 10 were combined with “or” | | | |
| 12  13  14 | HUMAN IMMUNODEFICIENCY VIRUS  ACQUIRED IMMUNODEFICIENCY SYNDROME  (HIV or AIDS or human adj1 immunodeficiency adj1 virus or autoimmune adj1 deficiency adj1 syndrome or acquired adj1 immune adj1 deficiency adj1 syndrome) | HUMAN IMMUNODEFICIENCY VIRUSES  ACQUIRED IMMUNE DEFICIENCY SYNDROME | HIV  ACQUIRED IMMUNODEFICIENCY SYNDROME | .TI or .TS |
| 15 | Set 12-14 were combined with “or” | | | |
| 17 | Set 11 and 15 were combined with “and” | | | |
| 18 | Set 17 was limited to 1990-present | | | |
| 19 | Set 18 was combined with country set using “and” | | | |
| 20 | Set 19 was limited to humans | | | |
| 21 | Set 20 was limited to English | | | |

All words in upper case words were searched as MeSH terms. All words in parentheses were searched as free text. **TI = title. TS = topic.
