# Supplementary material for: Systematic Review of TST Responses in People Living with HIV in Under-Resourced Settings: Implications for Isoniazid Preventive Therapy
Source: PLoS One. 2012 Nov 27;7(11):e49928. doi: 10.1371/journal.pone.0049928 (PMC3507950; doi:10.1371/journal.pone.0049928)
Supplement: Table S2 — Graded quality assessment checklist. (DOCX) [file pone.0049928.s002.docx]

**Table S2. Graded quality assessment checklist**

| **Quality assessment TOOL** | | | |
| --- | --- | --- | --- |
| ***Selection of the Subjects SCORE*** | | | |
| Age inclusion criteria is clearly stated (if not, age range is given) | 0 | 0.5 | 1 |
| The individuals selected are comparable in all respects with the source population (the population to which the results will be generalized) | 0 | 0.5 | 1 |
| The study indicates how many of the individuals asked to take part did so/analysed | 0 | 0.5 | 1 |
| The study indicates what percentage of individuals recruited didn’t return for TST reading | 0 | 0.5 | 1 |
| ***Assessment SCORE*** | | | |
| The study describes that patients with active TB were excluded | 0 | 0.5 | 1 |
| The study describes that symptom-based screening and/or clinical investigation were used to rule out active TB | 0 | 0.5 | 1 |
| The study describes that chest radiographs were used to rule out active TB | 0 | 0.5 | 1 |
| The study describes that sputum microscopy and/or culture was used to rule out active TB | 0 | 0.5 | 1 |
| The study describes the TST placement method (amount injected, trained staff, etc.) | 0 | 0.5 | 1 |
| The study describes how TST induration was read | 0 | 0.5 | 1 |
| The study describes if more than one reader for TST induration was used | 0 | 0.5 | 1 |
| The study describes the cutoff size used to determine TST-positivity | 0 | 0.5 | 1 |
| The study describes how the CD4 cell count was determined | 0 | 0.5 | 1 |
| The study describes that TST administration/reading and CD4 cell count were done at the same time | 0 | 0.5 | 1 |
| The study gives characteristics of study participants (including mean or median age) | 0 | 0.5 | 1 |
| The study indicates missing data for each variable of interest | 0 | 0.5 | 1 |
| **TOTAL NUMBER OF POINTS (MAX=16)** |  | | |
