# Supplementary material for: Systematic Review of TST Responses in People Living with HIV in Under-Resourced Settings: Implications for Isoniazid Preventive Therapy
Source: PLoS One. 2012 Nov 27;7(11):e49928. doi: 10.1371/journal.pone.0049928 (PMC3507950; doi:10.1371/journal.pone.0049928)
Supplement: Table S3 — Summary of studies meeting criteria for contacting authors for further data but for which insufficient secondary data was provided for study inclusion. (DOCX) [file pone.0049928.s003.docx]

**Table S3:** Summary of studies meeting criteria for contacting authors for further data but for which insufficient secondary data was provided for study inclusion.

| **Study** | **Country** | **Eligible subjects with TST placement (N)** | **Eligible subjects testing TST-positive (N)** | **Overall proportion of subjects testing TST-positive** |
| --- | --- | --- | --- | --- |
| Zhang et al (2010) [1] | **China** | **93** | **3** | 3.2% |
| Golub et al (2007) [2] | **Brazil** | **5492** | **1363** | 24.8% |
| Hawken et al (1997) [3] | **Kenya** | **595** | **136** | 22.9% |
| Kabeer et al (2011) [4] | **India** | **180** | **34** | 18.9% |
| Leidl et al (2010) [5] | **Uganda** | **89** | **42** | 47.2% |
| Talati et al (2011) [6] | **Zambia** | **298** | **128** | 43.0% |
| Toledo et al (2000) [7] | **Brazil** | **-** | **-** | - |
| Pistone et al (2002) [8] | **Senegal** | **204** | **48** | 23.5% |
| Rodriguez et al (1997) [9] | **Colombia** | **104** | **23** | 22.1% |
| Tegbaru et al (2006) [10] | **Ethiopia** | **116** | **47** | 40.5% |
|  |  |  |  |  |
| **Median proportion of study subjects testing TST-positive [%(IQR)] 23.5% (22.1-40.1%)** | | | | |

**References**

1. Zhang M, Wang H, Liao M, Chen X, Graner M, et al. (2010) Diagnosis of latent tuberculosis infection in bacille Calmette-Guerin vaccinated subjects in China by interferon-gamma ELISpot assay. Int J Tuberc Lung Dis 14: 1556-63.
2. Golub JE, Saraceni V, Cavalcante SC, Pacheco AG, Moulton LH, et al. (2007) The impact of antiretroviral therapy and isoniazid preventive therapy on tuberculosis incidence in HIV-infected patients in Rio de Janeiro, Brazil. AIDS 21: 1441-8.
3. Hawken MP, Meme HK, Elliott LC, Chakaya JM, Morris JS, et al. (1997) Isoniazid preventive therapy for tuberculosis in HIV-1-infected adults: results of a randomized controlled trial. AIDS 11: 875-82.

(4) Kabeer BS, Sikhamani R, Raja A. (2011) Comparison of interferon gamma-inducible protein-10 and interferon gamma-based QuantiFERON TB Gold assays with tuberculin skin test in HIV-infected subjects. Diagn Microbiol Infect Dis 71: 236-43.

(5) Leidl L, Mayanja-Kizza H, Sotgiu G, Baseke J, Ernst M, et al. (2010) Relationship of immunodiagnostic assays for tuberculosis and numbers of circulating CD4+ T-cells in HIV infection. Eur Respir J 35: 619-26.

(6) Talati NJ, Gonzalez-Diaz E, Mutemba C, Wendt J, Kilembe W, et al. (2011) Diagnosis of latent tuberculosis infection among HIV discordant partners using interferon gamma release assays. BMC Infect Dis 11:264.

(7) de Castro Toledo AC Jr, Greco DB, Antunes CM. (2000) Risk factors for tuberculosis among human immunodeficiency virus-infected persons. A case-control study in Belo Horizonte, Minas Gerais, Brazil (1985-1996). Mem Inst Oswaldo Cruz 95: 437-43.

(8) Pistone T, Kony S, Faye-Niang MA, Ndour CT, Gueye PM, et al. (2002) A simple clinical and paraclinical score predictive of CD4 cells counts below 400/mm3 in HIV-infected adults in Dakar University Hospital, Senegal. Trans R Soc Trop Med Hyg 96: 167-72.

(9) Rodriguez JI, Arias M, Paris SC, Arbelaez MP, Betancur J, Garcia LF. (1997) Tuberculin skin test and CD4+/CD8+ T cell counts in adults infected with the human immunodeficiency virus in Medellin, Colombia. Mem Inst Oswaldo Cruz 92: 245-50.

(10) Tegbaru B, Wolday D, Messele T, Legesse M, Mekonnen Y, et al. (2006) Tuberculin skin test conversion and reactivity rates among adults with and without human immunodeficiency virus in urban settings in Ethiopia. Clin Vaccine Immunol 13: 784-9.
